# Accuracy of Conventional and Machine Learning Enhanced Chest Radiography for the Assessment of COVID-19 Pneumonia: Intra-Individual Comparison with CT

**DOI:** 10.3390/jcm9113576

**Published:** 2020-11-06

**Authors:** Katharina Martini, Christian Blüthgen, Joan E. Walter, Michael Messerli, Thi Dan Linh Nguyen-Kim, Thomas Frauenfelder

**Affiliations:** 1 Institute of Diagnostic and Interventional Radiology, University Hospital Zurich, University of Zurich, 8091 Zurich, Switzerland; christian.bluethgen@usz.ch (C.B.); joanelias.walter@usz.ch (J.E.W.); thidanlinh.nguyen-kim@usz.ch (T.D.L.N.-K.); thomas.frauenfelder@usz.ch (T.F.); 2 Department of Nuclear Medicine, University Hospital Zurich, University of Zurich, 8091 Zurich, Switzerland; michael.messerli@usz.ch

**Keywords:** critical care, imaging CT/MRI, infection control, pneumonia, respiratory infection, viral infection

## Abstract

Purpose: To evaluate diagnostic accuracy of conventional radiography (CXR) and machine learning enhanced CXR (mlCXR) for the detection and quantification of disease-extent in COVID-19 patients compared to chest-CT. Methods: Real-time polymerase chain reaction (rt-PCR)-confirmed COVID-19-patients undergoing CXR from March to April 2020 together with COVID-19 negative patients as control group were retrospectively included. Two independent readers assessed CXR and mlCXR images for presence, disease extent and type (consolidation vs. ground-glass opacities (GGOs) of COVID-19-pneumonia. Further, readers had to assign confidence levels to their diagnosis. CT obtained ≤ 36 h from acquisition of CXR served as standard of reference. Inter-reader agreement, sensitivity for detection and disease extent of COVID-19-pneumonia compared to CT was calculated. McNemar test was used to test for significant differences. Results: Sixty patients (21 females; median age 61 years, range 38–81 years) were included. Inter-reader agreement improved from good to excellent when mlCXR instead of CXR was used (k = 0.831 vs. k = 0.742). Sensitivity for pneumonia detection improved from 79.5% to 92.3%, however, on the cost of specificity 100% vs. 71.4% (*p* = 0.031). Overall, sensitivity for the detection of consolidation was higher than for GGO (37.5% vs. 70.4%; respectively). No differences could be found in disease extent estimation between mlCXR and CXR, even though the detection of GGO could be improved. Diagnostic confidence was better on mlCXR compared to CXR (*p* = 0.013). Conclusion: In line with the current literature, the sensitivity for detection and quantification of COVID-19-pneumonia was moderate with CXR and could be improved when mlCXR was used for image interpretation.

## 1. Introduction

As the COVID-19 pandemic caused by SARS-CoV-2 spreads in the world, there is growing interest in the role and appropriateness of conventional chest radiographs (CXR) and computed tomography (CT) for management of patients with suspected or known COVID-19 infection. As the chest CT and CXR imaging pattern is non-specific and overlaps with other infections, the diagnostic value of imaging for COVID-19 is low and dependents upon radiographic interpretation. One study found that 56% of patients who presented within two days of diagnosis had a normal CT [1]. Conversely, other studies have identified chest CT abnormalities in patients prior to the detection of SARS-CoV-2 RNA. Given the variability in chest imaging findings, the American College of Radiology (ACR) does not recommend chest radiographs or CT alone for the diagnosis of or screening for COVID-19 [2]. Generally, the findings on chest imaging in COVID-19 are non- specific and overlap with other infections, including influenza, H1N1, severe acute respiratory syndrome (SARS) and Middle East respiratory syndrome (MERS) [3,4]. Therefore, detection of SARS-CoV-2 RNA is required, even if radiologic findings are suggestive of COVID-19 on CXR or CT [2].

Conventional radiography, however, plays a role in the detection and follow-up of lung changes in patients with COVID-19, and CT should be reserved for hospitalized, symptomatic patients with specific clinical indications such as the investigation for pulmonary embolism or other complications [1].

COVID-19 pneumonia, often presents as diffuse or patchy ground-glass opacities (GGO), which are less dense than classic consolidations, and therefore, the difference of density between normal lung tissue and the infectious process is less obvious and can be easily missed [1,5]. For this reason, the already relatively low sensitivity of CXR for the detection of consolidation (reported to be between 40 and 70% [5,6,7]) will be potentially even lower for COVID-19 pneumonia. Therefore, it would be desirable to have a post-processing tool, which has the capability to “enhance” the—at times—very subtle changes on CXR to make them more perceptive to the human eye. Further, one approach to increase the sensitivity for the detection of pulmonary consolidation and GGO on CXR is to improve the visual distinction between infiltrates and lung parenchyma and to eliminate overlying structures. Previous studies reported that the diagnostic accuracy of chest radiography can be improved by the use of Dual Energy radiography (DER) techniques [6]. In our study, we used a software tool based on machine learning postprocessing to generate new images, where lung processes are better delineated or “enhanced”. Machine learning enhanced CXR (mlCXR) could be a promising technique to accelerate the diagnosis and treatment of COVID-19 pneumonia, since lung lesions might be better and earlier visible on conventional imaging.

Accordingly, the purpose of this study was to compare the diagnostic accuracy of conventional CXR and mlCXR for the detection and quantification of the disease extent in COVID-19 patients compared to chest CT.

## 2. Experimental Section

### 2.1. Patient Population

The local ethics committee as well as the institutional review Board (IRB) approved the study and written informed consent was sought from all patients. Project number ID 2020-00092. In this retrospective cohort study, we included data from consecutive adult symptomatic patients with real time polymerase chain reaction (rt-PCR)-proven COVID-19 infection who have been admitted to our institution between March and April 2020. COVID-19 patients were diagnosed according to the “pneumonia diagnosis protocol for novel coronavirus infection (trial version 7)” [8] with two positive rt-PCR tests for COVID-19. To be included, patients needed to have conventional imaging (CXR and post-processed mlCXR) as well as a CT acquired within 36 h which served as standard of reference.

Electronic medical records served as source data for the collection of demographics, clinical, laboratory, and treatment data.

Since a high percentage of the COVID-19 population showed infective consolidation on CXR which would constitute in an unbalanced study population, we decided to add a control group with normal imaging studies: patients undergoing CXR and CT within 36 h from January 2014 to December 2015 were selected in order to be sure to have patients without COVID-19 infection.

### 2.2. Image Acquisition

#### 2.2.1. Conventional Chest Radiography

All patients underwent CXR in posteroanterior and lateral projection or anteroposterior projection at a tube current of 7mA and a tube voltage of 130kVp according to the institutions standard protocol. The mlCXR images were generated with the use of a commercially available software package (ClearRead Bone Suppression and Confirm, Riverrain Technologies, Miamisburg, US) which is a machine learning based software tool that generates two additional images: (1) an “enhanced image” where visual quality of the chest X-ray is optimized by increasing the conspicuousness of pulmonary structures and (2) a “bone supressed image” where bony structures such as the clavicles or the ribs are eliminated from the image.

#### 2.2.2. CT Protocol

Single-energy CT with or without intravenous contrast agent was performed in all patients on a third-generation CT scanner (SOMATOM Force, SOMATOM Definition AS, or SOMATOM Definition Flash; Siemens Healthcare; Forchheim, Germany) equipped with an integrated high-resolution detector (Stellar Technology, Erlangen, Germany; Siemens). Scanning parameters were as follows: CT was performed at 100 kVp with quality reference current-time product of 80 mAs, a pitch of 1.2, gantry rotation time 0.5 s, slice acquisition of 192 × 0.6 mm by means of a z-flying focal spot. The onsite CT technician detailed the breathing instructions to the patient.

All images were reconstructed with advanced modelled iterative reconstruction (ADMIRE, Siemens Healthcare, Forchheim, Germany) at a strength level of 3, using a slice thickness of 1.5 mm, an increment of 1 mm, and a tissue convolution kernel (Bl34). The image matrix was 512 × 512 pixels.

### 2.3. Image Analysis

The images were presented to two independent readers (attending radiologists, with 20 and 6 years of experience, respectively) in two reading rounds. In the first reading round, the CXR images were assessed. In the second reading round, readers had to assess the mlCXR images (both, enhanced and bone suppressed images), but not the CXR images. The second reading round was performed two weeks apart from the first one in order to lower the risk of a recall bias.

In both rounds, both readers independently assessed the images for the presence (yes/no) and disease extent (i.e., percentage of affected lung parenchyma) ((I) <25%, (II) 25–50%, (III) >50–75%, (IV) >75%) of pneumonia in the conventional images. To keep the readout as simple as possible, we chose to rate the overall percentage of affected lung parenchyma, and not the affected lung parenchyma in each single lobe. Estimation of disease extend was rated correctly when both CXR and CT estimates were in the same of the four disease extent categories. Additionally, readers had to assign confidence levels to their diagnosis on a 4-point score: (I) confident of true finding, (II) probable true finding, (III) probably no finding, and (IV) definitely no finding. Images were assessed in a random order over a time period of two days. In both reading rounds readers were blinded to the clinical information.

**Figure 1:**
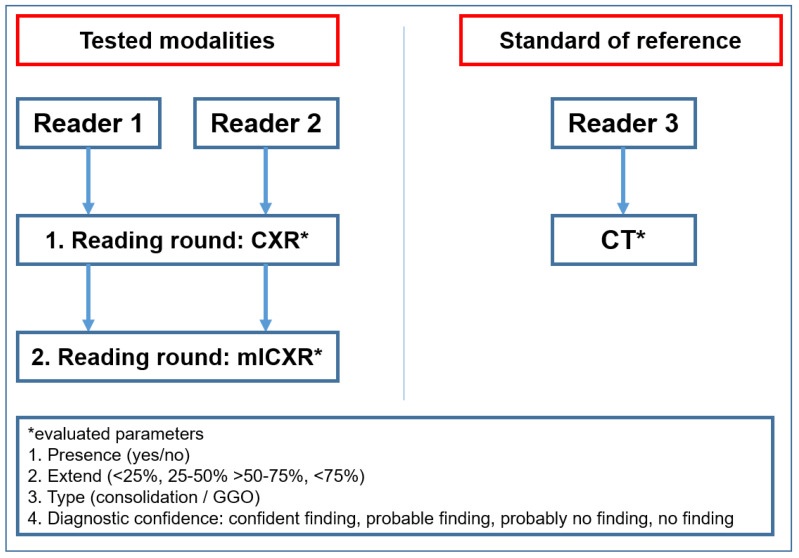
Image evaluation: Reader 1 and Reader 2 evaluated the conventional radiography images (CXR) and the machine learning conventional radiography (mlCXR). Reader 3 evaluated the standard of reference computed tomography images (CT). Evaluated imaging parameters were presence (yes/no) and extend and typo of parenchymal changes (consolidation or ground glass opacities (GGO)). Further, readers had to state the confidence level of their diagnosis. In all steps, images of *n* = 60 patients were evaluated.

CTs (which served as standard of reference) were read by a third reader (attending radiologist, with 20 years of experience in chest radiology) using the same classifications as for conventional radiography (presence (yes/no) and disease extent (I–IV). Additionally, the reader had to state the type of lung changes present on CT (i.e., classic consolidation vs. GGOs). If both, classic consolidation and GGOs, were present the reader had to state that both types were present. The reader was aware of patients’ symptoms but blinded to CXR and mlCXR diagnosis (Figure 1).

Analyses were performed using the picture archiving and communication system (PACS) of our hospital (Impax, Version 6.5.5.1033; Agfa-Gevaert, Mortsel, Belgium) on a high-definition liquid crystal display monitor (BARCO; Medical Imaging Systems, Kortrijk, Belgium).

### 2.4. Statistical Analysis

Statistical analyses were conducted using commercially available software (SPSS, release 26.0; SPSS, Chicago, IL, USA). Continuous variables were expressed as mean +/− standard deviation (SD) while categorical variables were expressed as frequencies or percentages.

Cohen’s Kappa (κ) was used to assess inter-reader agreement. Κ-results were stratified qualitatively by score (slight agreement 0.01–0.20; fair agreement 0.21–0.40; moderate agreement 0.41–0.60; good agreement 0.61–0.80; excellent agreement 0.81–0.99 [9]. Sensitivity and Specificity were calculated. McNemar test was used to investigate for significant differences between groups. A two-sided *p*–value below 0.05 was considered to indicate statistical significance.

## 3. Results

### 3.1. Patient Population

From March to April 2020, forty-one patients with rt-PCR-proven COVID-19 and nineteen control patients from January 2014 to December 2015 with CXR and CT within 36 h were retrospectively included in the study (21 females, 39 males; median age 61 years, range 38–81 years).

Patients in the control group underwent imaging for the following reasons: evaluation of the aorta (*n* = 2), infective consolidation (*n* = 10), search for metastasis (*n* = 5), trauma (*n* = 1), and pulmonary embolism (*n* = 1).

### 3.2. Clinical Findings in COVID-19 Patients

Mean time since onset of clinical symptoms was of 7.2 days (SD ± 8.9) at time of CT. Patients suffered from the following comorbidities: Cardiovascular disease (19.5%), arterial hypertension (31.7%), diabetes (26.8%), chronic renal dysfunction (22.0%), and chronic pulmonary disease (7.3%). Detailed information on clinical findings can be found in Table 1.

### 3.3. Imaging Findings

**Figure 2:**
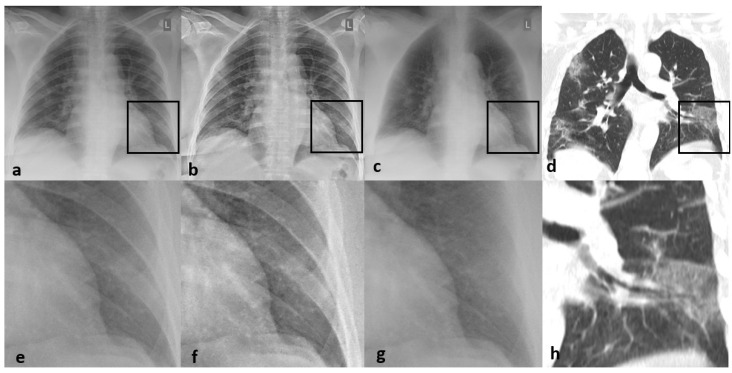
53-year-old male patient with rt-PCR proven COVID-19 pneumonia symptomatic since 6 days with fever and cough showing typical lung changes of COVID-19 pneumonia on (**d**,**h**) CT with wedge-shaped subpleural ground-glass opacification (GGO). These changes are hidden by the ribs in the (**a**,**e**) conventional radiograph (CRX), slightly visible on the (**b**,**f**) enhanced CRX, and better visible on the (**c**,**g**) enhanced CRX with bone suppression.

**Figure 3:**
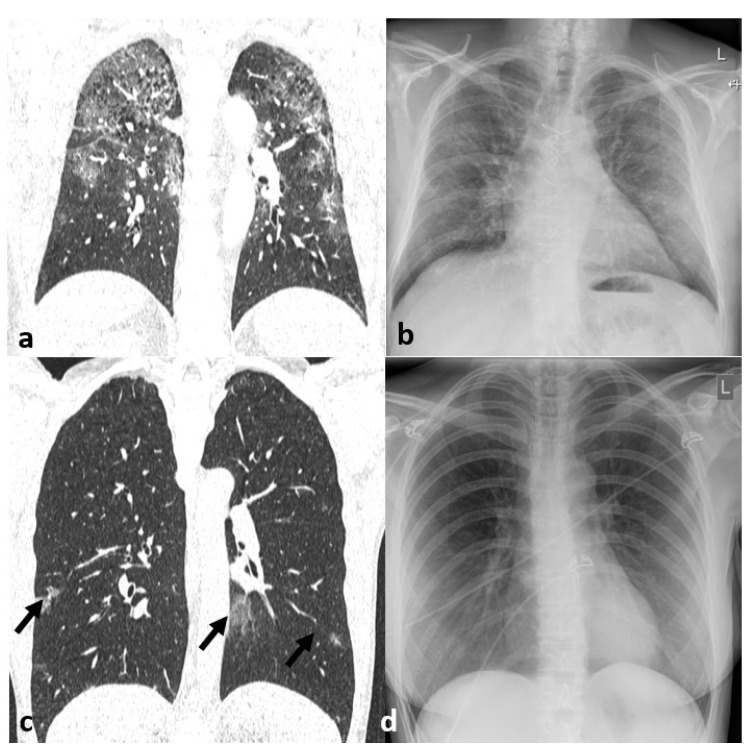
(**a**,**b**) 66-year-old male COVID-19 patient presenting with ground-glass opacification (GGO) predominantly in the upper lobes on (**a**) chest computed tomography comprising between 25 and 50% of the lung parenchyma. (**b**) Conventional radiography which was obtained 4 h before CT showed only subtle opacification in the left upper lobe and probably pneumonia affecting less than 25% of the lung parenchyma was given. (**c**,**d**) 55-year-old female COVID-19 patient presenting with subpleural ground-glass opacification (GGO) on (**c**) chest computed tomography (arrows) affecting less than 25% of the lung parenchyma. (**d**) Conventional radiography that was obtained 2 h before CT showed no suspicious changes and was rated as normal.

**Figure 4:**
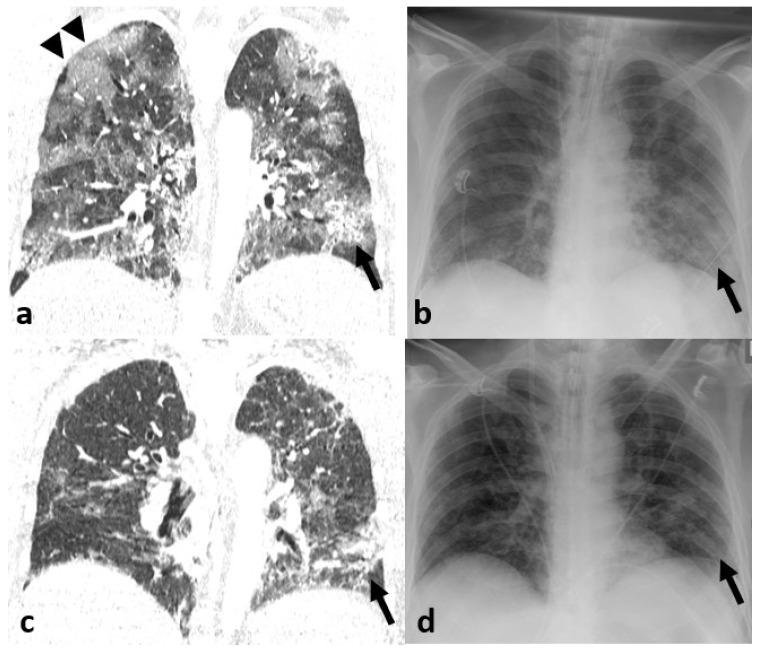
51-year-old male COVID-19 patient with baseline imaging (**a**,**b**) and follow-up imaging 21 days after (**c**,**d**). At baseline, diffuse ground-glass opacification (GGO) and consolidation in both lobes were present. While consolidation (arrow) is well appreciated on (**a**) computed tomography (CT) as well as on (**b**) conventional radiography (CXR), GGOs (arrowheads) were only visible on CT (arrowheads). In the follow-up image, regredient consolidation (arrow) in the left lower lobe can be appreciated on both (**c**) CT as well as (**d**) CXR, while changes in the extent of GGO were only appreciated on CT.

**Figure 5:**
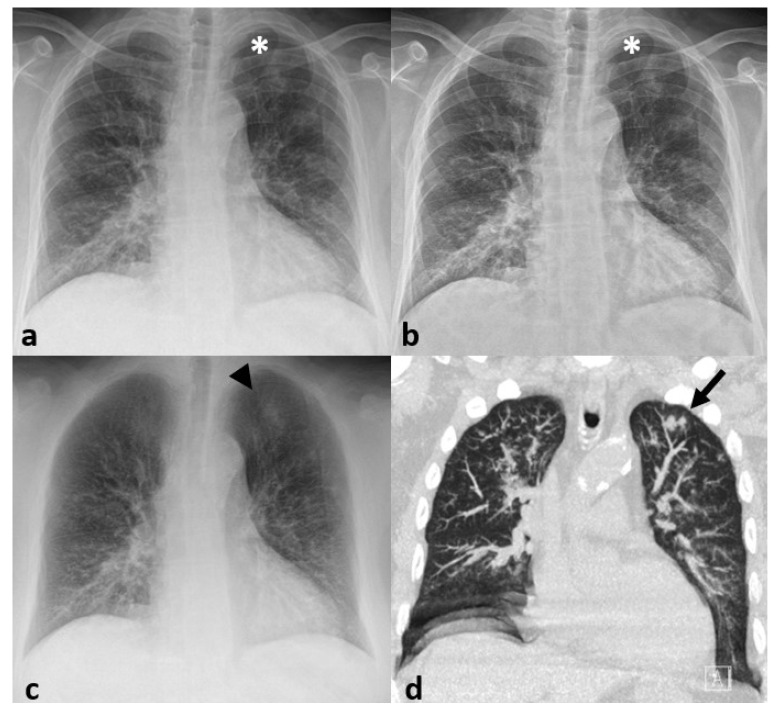
62-year-old male COVID-19 patient presenting with patchy consolidation in the left upper lobe on (**d**) chest computed tomography (arrow). These differences are not readily seen (**a**) in the conventional chest radiograph and (**b**) in the enhanced images (asterisks) and could also be due to a summation artifact of the ribs. Opacities become visible when bone was subtracted in the (**c**) bone suppressed images (arrowhead).

Thirty-nine out of 60 patients showed signs of pneumonia in form of consolidation or GGO on CT (65%). Inter-reader agreement improved from good to excellent when mlCXR instead of CXR was used (k = 0.831 vs. k = 0.742). The sensitivity and specificity for the detection of pneumonia on CXR was of 79.5% and 100%, respectively (Figure 2). Diagnosis was made in 66.7% with certainty, whereas in 33.3% of cases readers called their diagnosis a “probable” finding. Using mlCXR for image interpretation improved the sensitivity to 92.3% with a decline in specificity to 71.4%. The diagnostic confidence however increased from 66.7% to 86.7% (*p* = 0.013). Similarly, disease extent correlated better with enhanced CXR than with standard CXR (correct estimations in 77.3% of cases vs. 52.3% of cases), differences in the estimation of disease extent, however, were only tendencies (*p* = 0.590), Table 2 and Table 3, Figure 3. Altogether, sensitivity for the detection of consolidation was higher than that for the detection of GGO, Table 4, Figure 4 and Figure 5.

## 4. Discussion

The current COVID-19 pandemic calls for reliable imaging tools allowing for proper management of patients with SARS-CoV-2 infection frequently affecting the lungs. Non-contrast chest CT showed to have high sensitivity and specificity for the detection, severity assessment, and monitoring of COVID-19 associated lung changes [1,2,3,4], but has higher costs and is more difficult to implement (especially with patients who need to be properly isolated) than conventional radiography. Further, in some areas of the world, the access to CT might be restricted. Therefore, current guidelines advocate conventional radiography for the detection and follow-up of COVID-19 related lung changes, and CT should be reserved for hospitalized, symptomatic patients with specific clinical indications (i.e., ruling out pulmonary embolism or other complications).

CXR however, suffers some limitations such as a moderate diagnostic accuracy for the detection of pathologic lung changes compared to other imaging modalities such as CT [6,10] with sensitivities for the detection of infective consolidation ranging between 40 and 70% [5,6,7]. With GGOs, a typical pattern in COVID-19 pneumonia, the infectious process might be even less obvious compared to classic consolidations and is prone to be missed by the radiologist. Therefore, it would be desirable to have a postprocessing algorithm that is able to “enhance” pathologic findings on CXR to make them more perceptive to the human eye. In fact, in our study sensitivity for the detection of COVID-19 associated pneumonia could be improved from 79% on CXR to up to 92% when mlCXR images where used for image interpretation.

CXR is a projection-based imaging method, i.e., a three-dimensional structure is projected onto a two-dimensional image. Therefore, despite the high spatial resolution, CXR often lacks the possibility to differentiate structures with equal or similar density adjacent to each other or suffers from superposition of different structures [11]. The relatively low sensitivity of CXR is a known problem and in the last years different approaches have been used to overcome these shortcomings: One approach is Dual Energy Radiography (DER), where the radiologist instead of one image, obtains three images for evaluation: A soft tissue image and a bone image additional to the conventional X-ray image [12,13]. Martini et al. compared CXR with DER for the diagnosis of lung, mediastinal, and thoracic cage alterations and reported that DER had the greatest impact on the diagnosis of infectious and interstitial lung diseases increasing the sensitivity and inter-reader agreement [6].

While in DER more images have to be acquired in order to obtain the differently weighted images (at cost of higher dose), in the present study we used a machine learning based post-processing algorithm that enables the subtraction of structures that contain calcium (i.e., bone) in order to generate the “bone suppressed image” and a second image where pulmonary findings are enhanced. The advantages of the new method are that (1) no special equipment is needed, (2) the patient is not exposed to more radiation dose, and (3) no motion artifacts occur (a reported problem in DER from patient movement, breathing, or pulsation of the heart between the two acquisitions [4]).

COVID-19 pneumonia often presents as diffuse or patchy GGO [1,2,3,4], which are less dense than classic consolidation, and thus, the difference of density between normal lung tissue and the actual infectious process is less obvious and can so be easily missed by the radiologist. Especially, in cases were consolidation is interposed with GGO, the GGO part is prone to be overlooked and can lead to an underestimation of the disease extend.

The higher sensitivity observed with eCRX in our study came at cost of lower specificity. False positive findings are a well-known problem of all computer-aided detection (CAD) software, as the developers have to find the trade-off between high sensitivity and high specificity [14,15]. Something similar might be true for machine learning based software tools, which aim to enhance lung findings on images. An additional point might be that readers have to get familiar with the post-processed images in order to be able to discriminate true findings from “over-enhancement” of the software. Nevertheless, mlCXR resulted in an increase of diagnostic confidence. An accurate and reliable image-based detection, quantification of disease-severity, and progression is of great importance in determining the appropriate clinical management and respiratory support for infected patients [16]. In order to do that, the imaging modality used should monitor adequately the disease burden in evaluated patients. In our study, disease extent was only correctly quantified in about half of patients when CXR was used and increased to 77% when mlCXR was used; differences however, tended not to be statistically significant. This might be attributed to the small patient population and further investigations with bigger study cohorts might be needed for further evaluation.

The discrepancy in disease extent between CXR and CT might be mainly attributed to two factors. First, the physical properties of CXR in which structures of equal or similar density cannot be distinguished from each other. Second, the properties of COVID-19 pneumonia per se: (a) location in lung areas that are hidden and thus difficult to detect on CXR and (b) that COVID-19 pneumonia often consists in GGOs that are less visible on CXR. mlCXR is extremely powerful in difficult lung areas, where processes covered by overlapping structures (i.e., mediastinal shadow, ribs, and clavicles) and enhances lung processes—this could additionally explain the higher sensitivity for the detection and quantification of COVID-19 pneumonia on mlCXR.

Although chest radiography is considered not sensitive for the detection of pulmonary involvement in the early stage of COVID-19 pneumonia, in the current pandemic setting, chest radiography holds its position as useful diagnostic tool for monitoring rapid progression of lung abnormalities in COVID patients, particularly in intensive care units [16].

Noteworthy, we need to keep in mind that any sever viral pneumonia looks similar on imaging, which will pose a major problem with the upcoming flu season. The radiologist is not able to distinguish different viral entities with CXR or CT alone. Said this, rt-PCR remains the standard of reference in diagnosing COVID-19 infection.

Limitations of this study were as follows: Firstly, the retrospective setting and the relatively small sample size. Secondly, with imaging, we can only capture pathologic changes in the lung parenchyma, but we are not able to quantify lung function. Therefore, the amount and even distribution of infective consolidation on chest imaging in a given set of COVID patients might look similar but not necessarily correlate with clinical severity in all cases due to variations in baseline lung function. Thirdly, although there was a two-week washout period between reading the CXR and mlCXR images, it might be insufficient to completely exclude a recall bias. Fourthly, we cannot distinguish with the current study if the improved sensitivity of mlCXR was due to bone suppression or conspicuity enhancement since the two post-processed image datasets were simultaneously evaluated.

## 5. Conclusions

In conclusion, in line with the current literature, sensitivity for detection and quantification of COVID-19-pneumonia was moderate with CXR and could be improved when mlCXR was used for image interpretation. There is a tendency to underestimate the extent of COVID-19 changes in CXR, which should be taken into consideration for patient management when determining the therapy plan based on conventional imaging.

## Figures and Tables

**Table 1:** Clinical characteristics of study patients.

Female, *n* (%)	21 (35%)
Median age, years (range)	61 (38–81)
Infective consolidation, *n* (%)	39 (65.0%)
Median time interval CXR to CT, hours (range)	12 (0–36)
Mean time since symptom-onset, days	7.2 ± 8.9
Body mass index, *n* (%)	
≤25 kg/m^2^	6 (14.6%)
<25–30 kg/m^2^	10 (24.4%)
>30 kg/m^2^	25 (61.0%)
Cardiovascular disease, *n* (%)	8 (19.5%)
Arterial hypertension, *n* (%)	13 (31.7%)
Diabetes mellitus, *n* (%)	11 (26.8%)
Chronic renal dysfunction, *n* (%)	9 (22.0%)
Chronic pulmonary disease, *n* (%)	3 (7.3%)
Hepatitis or Liver cirrhosis, *n* (%)	3 (7.3%)
Malignancy, *n* (%)	6 (14.6%)
ARDS, *n* (%)	19 (46.3%)
Treatment type at diagnosis, *n* (%)	
Out of hospital	1 (2.4%)
In hospital	26 (63.4%)
ICU without mechanic ventilation	4 (9.8%)
ICU with mechanic ventilation	5 (12.2%)

CXR, Chest X-ray; CT, Computed tomography; ICU, Intensive Care Unit; ARDS, acute respiratory distress syndrome; *n*, number of patients.

**Table 2:** Imaging findings.

	CXR		mlCXR		CT
	Reader 1	Reader 2	Reader 1	Reader 2	Reader 3
Overall evaluated cases, (*n*, %)	60 (100.0)	60 (100.0)	60 (100.0)	60 (100.0)	60 (100.0)
Presence of pneumonia					
Overall, (*n*, %)	31 (51.7)	28 (46.7)	42 (70.0)	35 (58.3)	39 (65.0)
GGO, (*n*, %)	-	-	-	-	37 (61.7)
Classic consolidation, (*n*, %)	-	-	-	-	16 (26.7)
Extend of lung changes in cases with signs of COVID19-pneumonia					
<25%, (*n*, %)	1 (3.2)	3 (10.7)	6 (14.3)	5 (14.3)	2 (5.1)
25–50%, (*n*, %)	11 (35.5)	12 (42.9)	20 (47.6)	13 (37.1)	15 (38.5)
>50–75%, (*n*, %)	11 (35.5)	9 (32.1)	16 (38.1)	16 (45.7)	9 (23.1)
>75%, (*n*, %)	7 (22.6)	4 (14.3)	0 (0)	1 (2.9)	13 (33.3)

Conventional chest radiography (CXR), machine learning enhanced chest radiography (mlCXR), Computed tomography (CT), number of cases (*n*), Ground glass opacity (GGO).

**Table 3:** Diagnostic performance.

	CXR		mlCXR		
	Reader 1	Reader 2	Reader 1	Reader 2	*p*-Value
Diagnostic accuracy					0.031
Sensitivity, (95%CI)	79.5 (63–90)	71.8 (55–84)	92.3 (78–98)	89.7 (75–97)	
Specificity, (95%CI)	100.0 (81–100)	100.0 (81–100)	71.4 (48–88)	71.4 (48–88)	
PPV, (95%CI)	100.0 (86–100)	100.0 (85–100)	85.7 (71–94)	85.4 (70–94)	
NPV, (95%CI)	72.4 (53–87)	65.6 (47–81)	83.3 (58–96)	78.9 (54–93)	
Inter-reader agreement, kappa	0.834		0.805		
Diagnostic confidence					0.013
Overall certainty	40 (66.7)	36 (60.0)	52 (86.7)	50 (83.3)	
Overall un-certainty	20 (33.3)	24 (40.0)	8 (13.3)	10 (16.7)	
Def. COVID-19 pneumonia	25 (41.7)	21 (35.0)	37 (61.7)	37 (57.8)	
Probable COVID-19 pneumonia	9 (15.0)	13 (21.7)	8 (13.3)	9 (15.0)	
Probably no COVID-19 pneumonia	11 (18.3)	11 (18.3)	2 (3.3)	1 (1.7)	
Def. no COVID-19 pneumonia	15 (25.0)	15 (25.9)	12 (20.0)	13 (21.1)	
Accuracy of disease extent estimation					0.590
Exact estimation	23 (52.3)	19 (43.2)	33 (75.0)	34 (77.3)	
Under-estimation	16 (36.4)	21 (47.7)	14 (31.8)	17 (38.6)	
Over-estimation	5 (11.4)	4 (9.1)	7 (15.9)	4 (9.1)	
Inter-reader agreement, kappa	0.650		0.856		

Conventional chest radiography (CXR), machine learning enhanced chest radiography (mlCXR), positive predictive value (PPV), negative predictive value (NPV), definitely (Def.).

**Table 4:** Diagnostic accuracy for pattern recognition.

	CXR		mlCXR		Kappa
	Reader 1	Reader 2	Reader 1	Reader 2	
GGO on CT					0.669
Sensitivity, (95%CI)	37.5 (16–64)	40.0 (20–64)	61.7 (47–79)	67.6 (50–81)	
Specificity, (95%CI)	93.2 (8–98)	93 (80–98)	95.7 (76–99)	91 (70–98)	
PPV, (95%CI)	66.7 (31–91)	73 (39–93)	96.0 (78–99)	93 (74–99)	
NPV, (95%CI)	80.4 (66–90)	77 (63–87)	62.9 (45–78)	64 (45–79)	
Consolidation on CT					0.865
Sensitivity, (95%CI)	70.3 (53–84)	81.3 (54–95)	100 (77–100)	81.3 (54–95)	
Specificity, (95%CI)	95.7 (76–100)	91.1 (77–97)	98 (86–100)	90.9 (77–97)	
PPV, (95%CI)	96.3 (79–100)	76.5 (50–92)	94 (71–100)	76.5 (50–92)	
NPV, (95%CI)	66.7 (48–81)	93.3 (80–98)	100 (90–100)	93.0 (80–98)	

Conventional chest radiography (CXR), machine learning enhanced chest radiography (mlCXR), positive predictive value (PPV), negative predictive value (NPV).
